# Brief Eclectic Psychotherapy for Traumatic Grief (BEP-TG): toward integrated treatment of symptoms related to traumatic loss

**DOI:** 10.3402/ejpt.v6.27324

**Published:** 2015-07-06

**Authors:** Geert E. Smid, Rolf J. Kleber, Simone M. de la Rie, Jannetta B. A. Bos, Berthold P. R. Gersons, Paul A. Boelen

**Affiliations:** 1 Foundation Centrum '45/Arq Psychotrauma Expert Group, Diemen, The Netherlands; 2 Department of Clinical and Health Psychology, Utrecht University, Utrecht, The Netherlands; 3 Arq Psychotrauma Expert Group, Diemen, The Netherlands; 4 Department of Psychiatry, Academic Medical Center, Amsterdam, The Netherlands

**Keywords:** Grief, trauma, PTSD, depression, cognitive, attachment, brief eclectic psychotherapy, refugee, bereavement

## Abstract

**Background:**

Traumatic events such as disasters, accidents, war, or criminal violence are often accompanied by the loss of loved ones, and may then give rise to traumatic grief. Traumatic grief refers to a clinical diagnosis of persistent complex bereavement disorder (PCBD) with comorbid (symptoms of) posttraumatic stress disorder (PTSD) and/or major depressive disorder (MDD) following confrontation with a traumatic loss. Trauma survivors, who are frequently from different cultural backgrounds, have often experienced multiple losses and ambiguous loss (missing family members or friends). Current evidence-based treatments for PTSD do not focus on traumatic grief.

**Objective:**

To develop a treatment for traumatic grief combining treatment interventions for PTSD and PCBD that may accommodate cultural aspects of grief.

**Method:**

To provide a rationale for treatment, we propose a cognitive stress model of traumatic grief. Based on this model and on existing evidence-based treatments for PTSD and complicated grief, we developed Brief Eclectic Psychotherapy for Traumatic Grief (BEP-TG) for the treatment of patients with traumatic grief. The treatment is presented along with a case vignette.

**Results:**

Processes contributing to traumatic grief include inadequately integrating the memory of the traumatic loss, negative appraisal of the traumatic loss, sensitivity to matching triggers and new stressors, and attempting to avoid distress. BEP-TG targets these processes. The BEP-TG protocol consists of five parts with proven effectiveness in the treatment of PCBD, PTSD, and MDD: information and motivation, grief-focused exposure, memorabilia and writing assignments, finding meaning and activation, and a farewell ritual.

**Conclusion:**

Tailored to fit the needs of trauma survivors, BEP-TG can be used to address traumatic grief symptoms related to multiple losses and ambiguous loss, as well as cultural aspects of bereavement through its different components.

The loss of loved ones due to accidents, disaster, war, or criminal violence may bring about symptoms of persistent complex bereavement disorder (PCBD), such as persistent yearning/longing and distressing preoccupations regarding the traumatic nature of the death (APA, 2013). *Traumatic loss* refers to a situation where an individual is faced with the loss of one or several close family members or friends that occurred accidentally or in the context of war, homicide, suicide, or other situations of violence. Traumatic losses may be multiple and may include missing persons, also termed ambiguous loss (Boss, 2006). *Traumatic grief* refers to a clinical diagnosis of PCBD with comorbid (symptoms of) posttraumatic stress disorder (PTSD) and/or major depressive disorder (MDD) following confrontation with a traumatic loss. In patients who are referred for specialized treatment of complex psychological trauma symptoms, traumatic losses are common, especially in refugees (Nickerson et al., 2014). Contemporary PTSD treatments do not address grief specifically (Maercker & Znoj, 2010). Interventions designed for the treatment of PCBD mostly do not explicitly address comorbid disorders such as PTSD and/or MDD. However, in several studies, the efficacy of targeted treatment for PCBD has been demonstrated (Boelen, de Keijser, Van den Hout, & Van den Bout, 2007; Bryant, Kenny, & Joscelyne, 2014; Rosner, Pfoh, Kotoucìova, & Hagl, 2014; Shear, Frank, Houck, & Reynolds, 2005; Shear et al., 2014; Wagner, Knaevelsrud, & Maercker, 2006). Thus, there is a need to develop a treatment addressing both PCBD and PTSD/MDD, as a traumatic loss is a frequent cause of these disorders. We propose a cognitive stress model of traumatic grief that provides a rationale for the simultaneous treatment of symptoms of PCBD, PTSD, and MDD. Based on this model, we developed Brief Eclectic Psychotherapy for Traumatic Grief (BEP-TG) drawing from existing evidence-based interventions for PCBD and PTSD.

## Diagnostic considerations

The proposed DSM-5 diagnosis of PCBD (APA, 2013) comprises core symptoms as well as additional symptoms that are divided into two groups named reactive distress to the death and social/identity disruption. Core symptoms of PCBD include (1) persistent intense yearning and longing for the deceased, often experienced as recurrent pangs of grief, (2) intense sorrow and emotional pain in response to the death, (3) preoccupation with thoughts of the deceased, and (4) preoccupation with the circumstances of the death. The latter may manifest itself as distressing, intrusive thoughts regarding the traumatic nature of the death, often in response to loss reminders, including the deceased's last moments, degree of suffering and mutilating injury, or the malicious or intentional nature of the death (APA, 2013).

It must be noted that the PCBD has not yet been studied extensively (Boelen & Prigerson, 2012). That said, there is quite some evidence supporting the construct validity of prolonged grief disorder (PGD) (Prigerson et al., 2009), a condition resembling PCBD that includes persistent separation distress as well as difficulties accepting the loss and moving on without the lost person, that causes significant distress and disability at least 6 months following the loss. There is a large body of evidence showing that PGD symptoms are distinct from manifestations of uncomplicated grief, depression, PTSD, and other anxiety symptoms. PGD will likely be included in the forthcoming revision of the International Classification of Diseases (ICD-11; see Bryant, 2012; Maercker et al., 2013).

Simultaneous occurrence of PCBD (or its equivalent, PGD) with PTSD and/or MDD is common after traumatic loss. Comorbidity of these conditions has been observed in studies among, e.g., Western samples of individuals seeking treatment for bereavement-related distress (Simon et al., 2007) and community samples of elderly bereaved individuals (Newson, Boelen, Hek, Hofman, & Tiemeier, 2011). In addition comorbidity has been documented in non-Western samples. For instance, in victims of Rwandan genocide comorbidity of PGD, PTSD, and MDD was observed (Schaal, Dusingizemungu, Jacob, Neuner, & Elbert, 2012). And in a group of Cambodian survivors 30 years after the loss of a loved one during the Khmer Rouge regime (N = 775), 32% endorsed MDD, 11% endorsed PTSD, and 14% endorsed PGD (Stammel et al., 2013). In this study, the prevalence of PGD with comorbid PTSD and/or MDD was 12% (N. Stammel, personal communication, October 25, 2013).

PCBD, PTSD, and MDD include distinct as well as overlapping symptoms (APA, 2013). The onset and course of symptoms in relation to the loss may differ across these disorders. PCBD and MDD can share sadness, crying, depressed mood, loss of interest, and suicidal thinking. Following spousal bereavement, PCBD and MDD may be indistinguishable. However, the onset of chronic depression symptoms usually antedates the loss, and chronically depressed patients are more likely to show stress vulnerability, anxiety, marital dissatisfaction, and lack of instrumental support preceding the loss (Lotterman, Bonanno, & Galatzer-Levy, 2014). Symptoms that are not shared include a focus on the loss in PCBD, and loss of appetite, sleeplessness, and fatigue in MDD. PTSD may result from a traumatic event, such as witnessing or learning about the violent or accidental death of a family member or friend. Symptoms of PTSD include intrusions associated with the traumatic event, avoidance, negative changes in cognition and mood, and heightened arousal and reactivity. PTSD usually shows an acute onset within the first weeks or months following the loss, although delayed onset may occur in up to a quarter of cases of PTSD (Smid, Mooren, Van der Mast, Gersons, & Kleber, 2009). Both PTSD and PCBD can involve intrusive thoughts. Whereas intrusions in PTSD focus on the traumatic event, intrusive memories in PCBD mostly refer to moments with the lost person, accompanied by the pain associated with the irreversibility of his/her absence. Given the overlap in symptoms and frequent comorbidity, it is useful to combine treatment techniques addressing all three disorders.

## A rationale for the treatment of traumatic grief: a cognitive stress model

To provide a rationale for simultaneously targeting symptoms of PCBD, PTSD, and MDD, we present a provisional model that combines components of cognitive models of PTSD (Ehlers & Clark, 2000), MDD (Beck, 2008), and PCBD (Boelen, Van den Hout, & Van den Bout, 2006; Maccallum & Bryant, 2013; Rosner, Pfoh, & Kotoucìova, 2011) with elements of an attachment model (Shear et al., 2007) and behavioral model (Ramsay, 1977) of PCBD and a model of stress sensitization in PTSD (Smid et al., 2015).

Based on the proposed rationale for treatment, we developed BEP-TG for the treatment of patients with traumatic grief, comprising parts of a treatment protocol for PCBD (Boelen et al., 2007; Boelen & Van den Bout, 2011) and Brief Eclectic Psychotherapy for PTSD (BEPP) (Gersons & Olff, 2005; Gersons & Schnyder, 2013). BEPP is an effective form of treatment for PTSD (Gersons, Carlier, Lamberts, & van der Kolk, 2000; Lindauer et al., 2005) as well as comorbid MDD (Schnyder, Müller, Maercker, & Wittmann, 2011). The treatment rationale ties together the various treatment techniques included in BEP-TG. During BEP-TG, the therapist has to make decisions based on a clear and in-depth understanding of the processes that contribute to traumatic grief and that are amenable to treatment.

The model proposes that four processes contribute to the persistence and exacerbation of acute *separation and traumatic distress*, which includes yearning and pangs of grief, preoccupation, intrusive recollections, and despair that are all hallmark features of traumatic grief. These processes include:

 1. Inadequately integrating the memory of the traumatic loss;
 2. Negative appraisal of the traumatic loss;
 4. Sensitivity to matching triggers and new stressors;
 5. Attempting to avoid distress.
  The four processes are influenced by:
 6. Characteristics of the traumatic loss, attachments, and development;
 7. Cognitive processing and attachment reactions.

**Fig. 1:**
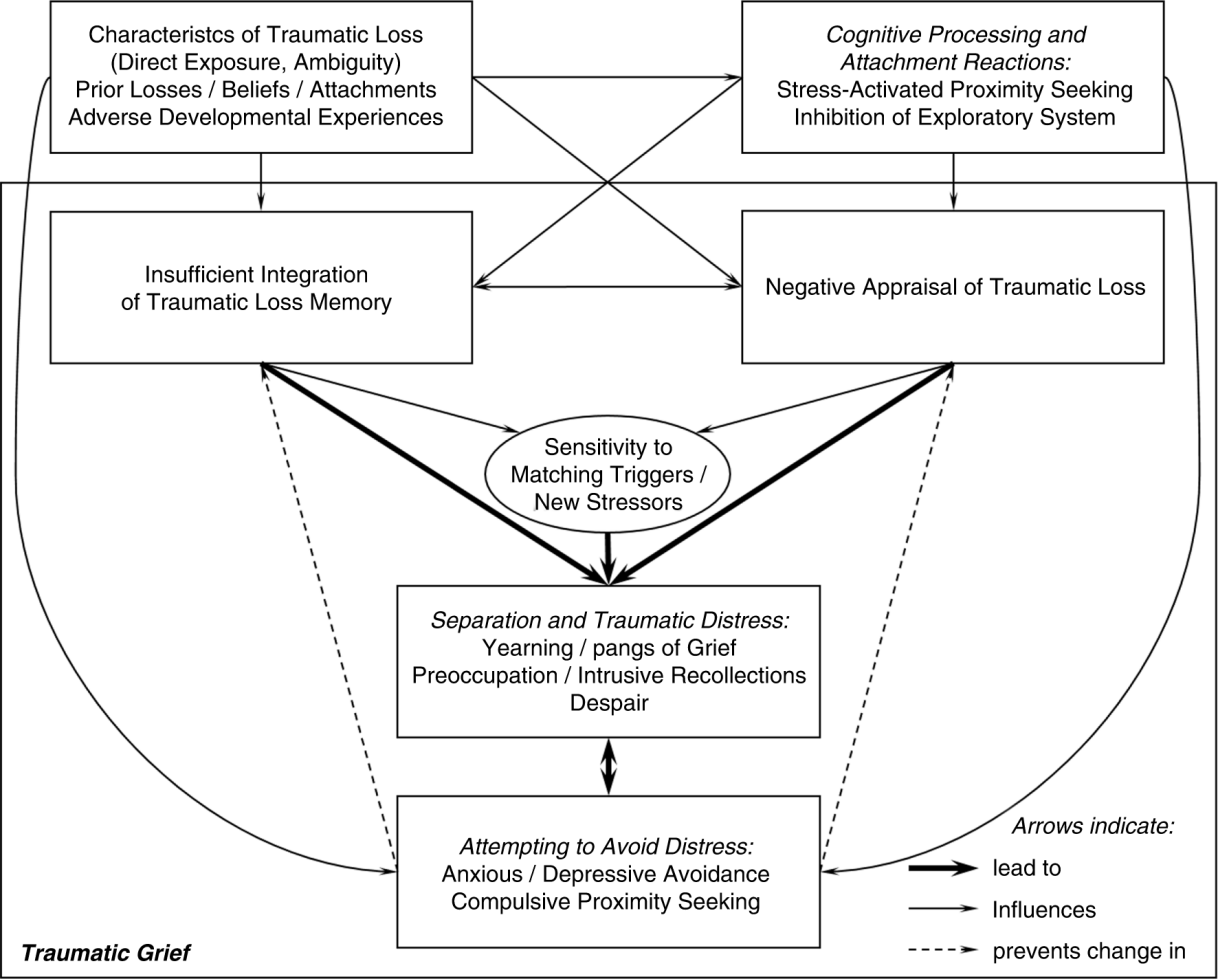
A cognitive stress model of traumatic grief.

A graphical representation of the model is shown in Fig. 1.

### 1. Inadequately integrating the memory of the traumatic loss

The model proposes that, in traumatic grief, the memories of the traumatic loss are poorly elaborated and inadequately integrated into their context in time, place, subsequent, and previous information and other autobiographical memories (Boelen et al., 2006; Ehlers & Clark, 2000). The factual knowledge that the separation is irreversible fails to get linked with long-term memory content about the self and the relationship with the lost person (Boelen et al., 2006). The memories of the lost person as well as the events leading up to the death lack context in time, and consequently retain their “here and now” quality, yielding a sense of persistent separation and traumatic distress.

### 2. Negative appraisal of the traumatic loss

A traumatic loss may present individuals with information that violates previously held global beliefs about the self, life, and the future. The traumatic loss may strengthen or induce maladaptive cognitions regarding safety, trust, power, esteem, and intimacy (McCann, Sakheim, & Abrahamson, 1988). Catastrophic interpretations of one's own grief reactions may be important, for example, interpreting intense emotional reactions as intolerable, insane, or as signifying a loss of control. Self-blame and negative views of responses of the social environment may lead to feelings of guilt and anger. Self-blame may be especially prominent if the death is felt as a failure of caregiving, such as following suicide or after the death of a child (Shear et al., 2007). Thus, negative appraisals of the traumatic loss contribute to persistent separation and traumatic distress.

### 3. Sensitivity to matching triggers and new stressors

*Matching triggers*, i.e., trauma and grief reminders may evoke or exacerbate separation and traumatic distress by reactivating warded off memories of the traumatic loss and/or the deceased (Ramsay, 1977; Rosner et al., 2011). Continuous searching for the deceased may lead to strong perceptual priming and a high likelihood of finding matching triggers. In addition, *new stressful life events* may be perceived as more stressful due to negative schemas that produce an attentional bias, negatively biased interpretations, and depressive symptoms. Stressful life events may include interpersonal tensions within bereaved families that may also be common after ambiguous loss (Boss, 2006). Although severe life events (such as the death of a loved one) are common precipitants of depression, milder stressful life events may provide an alternate pathway to depression in vulnerable individuals. Indeed, the triggering events of successive episodes of depression become progressively milder, suggesting a sensitization effect (Monroe & Harkness, 2005). Similarly, in PTSD, stress sensitization has been shown to play a role in its progression (Smid et al., 2015). Inclusion of stress sensitization in the model serves to explain the often fluctuating nature of separation distress (Schut, de Keijser, Van den Bout, & Dijkhuis, 1991) and traumatic distress (Smid et al., 2009, 2015) over time.

### 4. Attempting to avoid distress

Attempting to avoid separation and traumatic distress may be in part both consequence and cause of the unacceptability of the loss and the intrusive, unwanted character of the traumatic memories.

#### Anxious avoidance strategies

Anxious avoidance strategies occur when individuals believe that confronting the reality of the traumatic loss will lead to madness, loss of control, or otherwise unbearable consequences (Boelen et al., 2006). Examples include thought suppression, avoidance of loss reminders, and rumination (Eisma et al., 2013) about how the traumatic loss could have been prevented, or about how revenge can be achieved. Anxious avoidance may impede the elaboration and integration of the traumatic loss memory.

#### Depressive avoidance strategies

Depressive avoidance strategies occur when individuals engage in inactivity and withdrawal, and refrain from activities that could provide positive reinforcement and that were important prior to the loss (Boelen et al., 2006). Examples include avoiding activities that were shared with the deceased. Feelings of failure as a caregiver can lead to restriction in specific kinds of satisfying activities to avoid feelings of guilt (Shear et al., 2007). Depressive avoidance strategies may prevent a change in the appraisal of the traumatic loss.

#### Compulsive proximity seeking

Compulsive proximity seeking occurs when individuals spend inordinate amounts of time in imaginal company with the deceased person (Boelen et al., 2006; Shear et al., 2007). Examples include spending long hours at the cemetery or looking at pictures, constructing memorials, talking to or about the lost person as if he/she were still alive, cultivating particular mourning rituals or seeking comfort through memories. Turning away from the reality of the loss contributes to traumatic and separation distress when individuals fear that confronting this reality will have disastrous consequences (Boelen et al., 2006).

### 5. Characteristics of the traumatic loss, attachments, and development

#### Direct exposure and ambiguity

Direct exposure to horrific details of the traumatic loss of a loved one likely increases the risk of traumatic grief. Negative appraisals of the loss are likely to be influenced by horrific details. Generally, violent deaths are more likely to generate distressing intrusive memories than nonviolent deaths (Boelen, de Keijser, & Smid, 2014). Ambiguity, i.e., a lack of information such as with missing persons (ambiguous loss) may also be associated with increased distress, because the situation cannot be resolved, destroying the individual's sense of mastery (Boss, 2006).

#### Prior losses, beliefs, and attachments

Prior experiences with loss may become linked with the current traumatic loss and thereby increase its negative meaning. A traumatic loss can disrupt preexisting strongly positive beliefs about the self, such as the belief that no one can ever harm oneself, or reinforce preexisting negative beliefs and lack of trust in oneself or the world. Moreover, the attachment style of the individual and the nature of the attachment relation may shape the grief reaction (Maccallum & Bryant, 2013). Attachment style may impact on the efficacy of disclosure in treatment (Stroebe, Schut, & Stroebe, 2006). Specifically, disclosure may be more effective in individuals with insecure compared with secure attachment styles.

#### Adverse developmental experiences

Adverse developmental experiences foster negative attitudes and biases about the self, which are integrated into the cognitive organization in the form of schemas (Beck, 2008). The meaning of early losses and other adverse events is transformed into a durable attitude (e.g., helplessness), which may be activated by the traumatic loss (Beck, 2008). Attempting to avoid distress may be influenced by adverse developmental experiences as well as prior losses, beliefs, and attachments.

### 6. Cognitive processing and attachment reactions

If the individual lacks conceptual processing and engages mainly in data-driven processing (i.e., processing the sensory impressions), which is more common in children and people with low intellectual ability, then the trauma memory will be relatively difficult to retrieve intentionally (Ehlers & Clark, 2000). Data-driven processing may preclude a self-referential perspective while experiencing the traumatic loss, and the lack of this perspective may prevent the integration of the loss into the continuum of other autobiographic memories in time (Ehlers & Clark, 2000). Attachment related automatic responses influence cognitive processing of the traumatic loss. The stress of bereavement activates attachment proximity seeking (Shear et al., 2007). Strong activation of attachment proximity seeking is associated with inhibition of the exploratory system, resulting in loss of interest in the world and inhibition of goal seeking (Shear et al., 2007). Inhibition of the exploratory system may thus contribute to depressive avoidance strategies.

## Treatment of traumatic grief

The 16-session BEP-TG protocol consists of three stages with different emphasis on processes and influencing factors of the cognitive stress model. First, the *Information and motivation* stage (one session) provides an introduction to the treatment for the patient and close others. During this stage, characteristics of the traumatic loss are discussed and basic information is provided on cognitive processing and attachment reactions. Second, the *Grief-focused exposure, writing assignments and mementos* stage (five sessions) specifically targets two core processes of the model: inadequately integrating the memory of the traumatic loss, and attempting to avoid distress. Third, the *Finding meaning, activation, and farewell ritual* stage (10 sessions) additionally targets negative appraisal of the traumatic loss and sensitivity to matching triggers and new stressors.

BEP-TG is indicated in patients with traumatic grief, who express a wish for help in coping with the loss. BEP-TG is contra-indicated in patients diagnosed with psychotic depression, cognitive impairment, psychotic disorders, bipolar disorders, substance dependence, and severe personality or eating disorders. The aim of treatment is learning to live with the loss (or in case of ambiguous loss, with ambiguity) and relieving symptoms of PCBD, PTSD, and/or MDD.

**Fig. 2:**
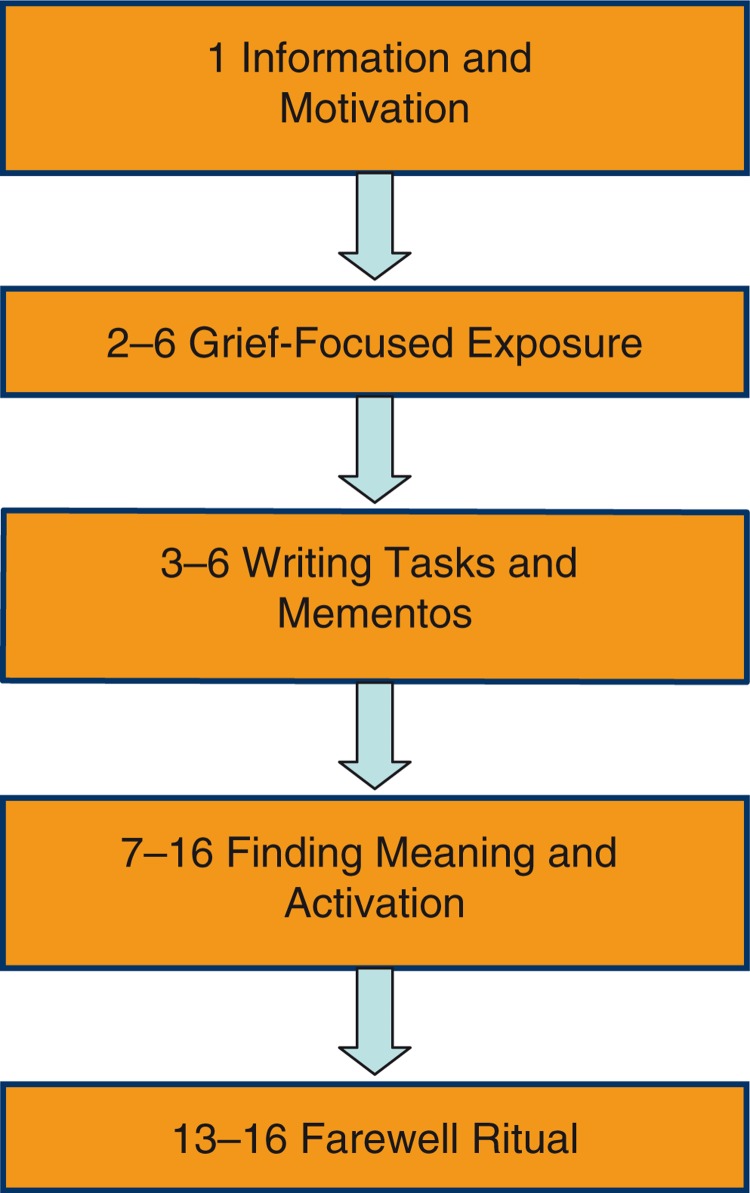
BEP-TG overview. Numbers indicate session numbers.

Across the three stages, the BEP-TG protocol consists of five components. Figure 2 shows an overview of the BEP-TG components, also indicating the planning of the BEP-TG components across the 16 sessions. Due to its emphasis on cognitive aspects (e.g., forms of finding meaning and assignments to create a narrative of the traumatic experience) and due to its concrete elements (e.g., mementos and rituals) BEP-TG can accommodate cultural aspects of grief. The characteristics of grief have been studied in various cultures (Rosenblatt, 2008). Meaning making occurs in the interchange between the individual and the culture. Indeed, an individual seeks to make sense of his or her experience using cognitive or mental models that are supplied by that individual's culture.

BEP-TG can be used in traumatic grief following multiple loss as well as following ambiguous loss (missing persons). In the next paragraphs, this will be illustrated using a case vignette.

## Description of BEP-TG components

*Jack, now 25 years old, from Liberia, lives undocumented in the Netherlands. He was referred for treatment because of PTSD and comorbid depression. At age 14, his house was attacked by the rebels. His beloved grandmother burned in the house, while lying paralyzed on her bed. Jack wasn't at home; at that time he was forced to be a child soldier. Mother, brothers and sisters are missing and most likely deceased. (Father left the family when Jack was a small child.) Jack has recurrent nightmares in which his grandmother is appearing with a suitcase in her hand, staring at him. Jack's interpretation is that she accuses him of her death, because he wasn't there to protect her. He thinks her soul cannot find rest because there had been no burial ritual. He wants to accept her death but doesn't know how. Jack experiences intense distress in response to reminders of traumatic events, as well as dissociative reactions (depersonalisation and derealisation). He tries to avoid thoughts and memories of being a child soldier, the way his grandmother died, as well as his missing mother and sibs. Jack reports a depressed mood, increased irritability, loss of interest in former activities, social withdrawal, difficulty concentrating, and suicidal thoughts. He is clinically diagnosed with PCBD with traumatic loss, PTSD with dissociative symptoms and delayed expression, and MDD, moderate, according to DSM-5. On the Clinician-Administered PTSD Scale (Weathers, Keane, & Davidson, 2001) his score is 92, indicating extreme PTSD. On the Traumatic Grief Inventory, a self-report measure of PCBD (Boelen and Smid, unpublished), he indicates that he has suddenly emerging thoughts and images of grandmother's death, he feels strong longing for her, he feels confusion about his role in life, he has difficulty accepting her death, he avoids thoughts that are reminding him of her death, he feels anger about her death, moving on with his life is difficult, his life feels empty or meaningless without her, he has strong self-blaming thoughts about her death, and his functioning has deteriorated seriously as a result of her death*.

### 1. Information and motivation

*Jack was asked to bring in his best friend at the start of therapy. Jack, who has no family in the Netherlands, doesn't want to bother his friend. The therapist decides to respect this. Jack recognizes much of the information provided about grief reactions. Explanation of the treatment: gradual exposure to the images Jack tries to avoid as much as he can of the murder of his grandmother. Expression of emotions is encouraged*.

At the beginning of the treatment we confront the patient, who is then preferably accompanied by a partner or close friend, with a view on his or her symptoms as related to traumatic grief. Examples of therapist explanations are shown in Table 1. After this, the aim of the treatment is explained: learning to live with the loss and relieving symptoms related to grief, posttraumatic stress, and depression. In case the patient has experienced multiple losses, the loss of a loved one currently perceived as most painful is the focus of therapy. The therapist notes the presence of ambiguous loss. The further course of treatment is briefly explained.

**Table 1:** Examples of therapist explanations and questions

BEP-TG component	Aim	Example
Information and motivation	Explaining traumatic grief symptoms	When we lose a loved person in a traumatic situation, we are often forced to remain active and not to dwell on our feelings. Painful feelings about the loss can be so severe that one may become afraid of them. Therefore, people may try to control their feelings or to avoid them. This costs a lot of energy: people become exhausted and forgetful. Therefore, one needs to allow one's feelings of grief and sadness, in order to accept finally that the death has occurred.
Grief-focused exposure	Explaining different types of avoidance	Some people: …suppress thoughts and feelings about the loss, because they find it just too painful to realize what the loss means to them, and sometimes because they are afraid that allowing these feelings will turn them crazy; …tuck away or suppress memories of certain events, because they are intensely painful; …avoid certain places, such as the tomb of the deceased or objects, such as photos of the deceased; …keep trying to be with the deceased and stick to various rituals surrounding the lost person.
	Evoking memories and feelings	What do you miss most now that he is dead? At what times do you miss him? What do you feel when it comes to your mind that you will never will be able to do your favorite activities with him anymore? Remember when you have spoken for the last time with the deceased. Describe what you see. How do you feel when you recall that memory?
Finding meaning and activation	Explaining activation	The traumatic loss of a loved one often puts a brake on all sorts of activities. Life is “on hold.” To process a loss, it is important that we once again undertake activities that provide pleasure and meaning. You then gradually can get back to undertaking activities that are important to you. When you continue to do things that you always did, you will have a chance that positive experiences will be a counterweight to all the sadness and help you through the process. How would you like your life to be in the near future, taking into account this loss? Imagine that it is now a year later, you have come to terms with the loss and you are able to undertake enjoyable and satisfying activities. What would your life be like? What would you do?

### 2. Grief-focused exposure

*Jack tells loving memories about his grandmother; how much he misses her as well as his mother. Jack tells about the last time he saw his grandmother, with lots of tears. Jack tells about the moment he heard about her death. Gradual exposure to the way he imagines his grandmother was burned then follows, very detailed*.

Prior to the start of the exposure, the therapist explains as far as necessary that people with traumatic grief can avoid their pain related to the loss. The therapist explains that avoidance of painful memories can be achieved in different ways. Therapist explanation examples are provided in Table 1. The therapist identifies the main type of avoidance of the patient and then selects the appropriate grief-focused exposure variant.

#### 2.1. General exposure

In patients who find it difficult to accept the loss or feelings of sadness, general exposure is the appropriate exposure variant. The aim is to enable the patient to realize that he can stand the painful reality of the loss and the feelings and thoughts associated with it. The therapist encourages the patient to reflect on the loss, asking about the deceased person and the circumstances surrounding the loss and noticing (nonverbal) emotional reactions of the patient. Then the therapist encourages the patient to get to emotionally charged memories. The patient is asked to articulate what he misses most, now that his loved one is gone and to verbalize his thoughts and feelings on realizing that the deceased person never will come back.

#### 2.2. Imaginal exposure

When a patient avoids particular memories of specific events surrounding the loss, imaginal exposure will be the preferred exposure variant. The aim of this type of exposure is a reduction of emotional responsiveness, through habituation and cognitive change. The therapist asks the patient to tell the event, at first globally and preferably chronologically. The patient is then asked to close his eyes and focus on the most distressing moments surrounding the traumatic loss. The therapist encourages the patient to tell what he sees, hears, smells, feels, and thinks in the present tense. Imaginal exposure is continued until recollections of the loss arouse less intense emotions and are no longer avoided. It is strongly facilitated by making audio recordings of the exposure sessions and instructing the patient to listen to the recording every day.

#### 2.3. Stimulus exposure

When a patient avoids specific situations or objects that remind him or her of the traumatic loss, stimulus exposure provides an opportunity for the patient to test fearful expectations and to find these disconfirmed. First the therapist will determine a fear hierarchy together with the patient to enable the exposure tasks to be performed in gradual steps. With a patient who is afraid to visit the grave of the deceased, the therapist can start exposure with the imagination of visiting the grave; then ask the patient to visit the grave with a relative or friend; and finally encourage the patient to visit the grave by himself.

#### 2.4. Diminish mourning behavior

When a patient keeps trying to be with the deceased and sticks to various rituals surrounding the lost person with the aim of avoiding the reality of the loss and the associated feelings (e.g., spending long hours at the cemetery or with belongings of the deceased every day) diminishing mourning behavior is indicated. Diminishing dysfunctional mourning behaviors can be achieved through response prevention. The therapist explores in which way the patient tries to continue the relationship with the deceased. Patients may be reluctant to reduce mourning behavior because of feelings of guilt or betrayal. In motivating the patient to reduce the mourning behavior, it can be helpful to ask the patient how the deceased person would want the patient to behave. The therapist helps the patient to understand that remembering a deceased loved one does not preclude finding a way to get on with life.

### 3. Mementos and writing assignments

*Jack starts writing letters. The first letter is a letter to his grandmother. He asks her to forgive him and how much he is missing her. He writes in a park on a quiet spot. He cries a lot. The second letter is a letter to the rebels. He expresses a lot of anger. Jack draws the house where he lived with his mother and grandmother, as well as grandmother's room. He has no mementos of this period in his life*.

During the first phase of treatment, bringing mementos to the sessions and writing letters are useful alternatives for avoidance of feelings of sadness or anger associated with the traumatic loss.

#### Mementos

Mementos are objects that symbolize the bond with the deceased, such as clothing, songs, or pictures, or objects that are (symbolically) linked to the traumatic event. Looking at and talking about these objects will bring the deceased close to mind. The meaning of the object and the feelings it brings about are discussed. Some mementos can also be used for the farewell ritual, described below. In case there are no mementos, which is often the case in refugees, the therapist can make use of drawings made by the patient.

#### Writing assignments

Writing assignments are also a useful tool to enable patients to go beyond avoidance of emotions and to promote cognitive change (Wagner et al., 2006). There are different types of writing assignments. Table 2 shows different types of writing assignments as well as the reasons for choosing specific assignments in specific clinical situations.

**Table 2:** Writing assignments

Writing assignment	In patients who …	Description
Ongoing farewell letter	Have difficulty allowing feelings of sadness	A letter to the deceased in which the patient writes what he has always wanted to say to the deceased, what he misses most, expressing his longing for the deceased
Three letters task	Are very ambivalent about the deceased	In a first letter, negative feelings toward the deceased are verbalized (anger, discontent); in a second letter, positive feelings are expressed(love, things that are missed); in a third and final letter positive and negative feelings are integrated
Letter to an imaginative companion in misfortune	Have strong maladaptive cognitions	A letter to a non-existing person who has been through exactly the same loss, trying to help this imaginary companion to re-evaluate maladaptive cognitions about the loss
Angry letter	Have difficulty dealing with feelings of anger	A letter to a perpetrator of murder, negligent bystanders, government, or other agency that is held responsible, in which uncensored anger, including insults and diatribes can be expressed. The letter is not sent. Sometimes, burning the angry letter is part of the farewell ritual.

### 4. Finding meaning and activation

*Despite housing problems, Jack continues treatment. He engages in an imaginal conversation with grandmother. Grandmother is sitting on an empty chair and forgives Jack. She tells him that he may continue his life. Jack also imagines that he is a judge at the international court, where he sends the rebels to jail. Jack now realizes that he wants to live again. He used to be interested in sports, especially in soccer. He starts taking part in street soccer. He wants to take better care for his body and starts doing push-ups. Jack and his best friend decide to have dinner together more often*.

#### Finding meaning

Experiencing a traumatic loss confronts people with vulnerability, helplessness, and often extreme and frightening aspects of human behavior. The traumatic loss as well as intervening stressors impact on themes of safety, trust, intimacy, esteem, and power (McCann et al., 1988). The patient needs to develop a more relative and realistic sense of safety, to explore how to trust others again, to overcome emotional barriers to being intimate again with others, to mitigate the effects of guilt or shame on self-esteem, and to regain sufficient sense of control despite vulnerability, anger, or feelings of revenge.

The role of the therapist is often to acknowledge and empathize with the thoughts and feelings regarding these themes, as well as to identify, challenge, and modify negative assumptions. Strong feelings of guilt can be addressed using cognitive techniques that may help discriminating between real and perceived (exaggerated) guilt, such as the “responsibility pie” (Greenberger & Padesky, 1995), in which the contribution of the self and others to the traumatic loss can be visualized. The therapist may guide an imaginal conversation with the person who died, in which the patient talks to the deceased person, and also answers (Shear et al., 2005). This technique may also mitigate guilt feelings. When working with non-Western patients, a culture sensitive attitude is essential, and special attention needs to be given to rituals that could not take place.

#### Activation

When the patient has been able to develop a more realistic sense of safety, he will be more capable of looking ahead and shift his focus from threat to possibility. The therapist explores what kind of activities a patient undertakes and to what extent these activities are satisfactory. What has changed since the traumatic loss? The therapist challenges the patient to look ahead and encourages the patient to think of important social, recreational, and, if relevant, work-related goals in the near future.

### 5. Farewell ritual

*Before the last treatment session, Jack burns his letters to the rebels. Jack carries out a plan that he had developed to organize a burial ritual to be performed at the mosque. In honor of his grandmother, there was recitation of texts from the Koran, prayers together with old men, and a burial meal*.

Farewell rituals have been used traditionally in funerals where the body of the deceased was not present. For example, when a fisherman had drowned at sea, possessions of the fisherman were used instead of the body (Gersons & Schnyder, 2013). A farewell ritual facilitates creating personal, adaptive meaning in different ways, for example, by symbolizing an irreversible transition, justifying emotions, facing the reality of the loss, learning from experience, and experiencing support and comfort (Van der Hart & Boelen, 2003). The farewell ritual symbolizes a revised attachment bond with the deceased: the memory of the deceased may still be cherished, but the deceased is no longer symbolically kept alive (Van der Hart & Boelen, 2003). The patient designs a farewell ritual that he finds appropriate. Examples of a farewell ritual include: visiting a special place; creating a symbol or lieu of remembrance; performing a culturally appropriate ritual; renouncing things related to the traumatic circumstances of the death; burning the angry letter. The therapist is not present at the ritual. The ritual also implies a departure from the therapist and a reunion with loved ones, therefore the patient is encouraged to share the farewell ritual with a partner or a close friend.

## Concluding remarks

*Jack tells that he feels peace at heart. The nightmares disappear. He feels less tension about his grandmother, as well as his missing mother and sibs. He reports feeling better by 70%. His CAPS total score drops from 92 pre-treatment to 77 post-treatment, a clinically significant improvement (Weathers et al*., 2001).

Traumatic grief may develop in bereaved individuals following the traumatic loss of a loved person. A cognitive stress model of traumatic grief provides a rationale for treatment. According to this model, processes contributing to traumatic grief include inadequately integrating the memory of the traumatic loss, attempting to avoid distress, negative appraisal of the traumatic loss, and sensitivity to matching triggers and new stressors. BEP-TG aims at elaborating and integrating memories of the traumatic loss, providing alternatives for avoidant strategies to control distress, modifying negatively biased thinking, developing a realistic sense of safety and revising the relationship with the lost person. BEP-TG simultaneously targets separation and traumatic distress as well as other symptoms of PCBD, PTSD and MDD. Thus, BEP-TG provides integrated targeting of symptoms related to traumatic loss that distinguishes it from existing treatments for PCBD, PTSD, or MDD. As illustrated by the case vignette, BEP-TG may reduce symptoms of traumatic grief despite chronicity, severe ongoing stressors, cultural differences, and multiple as well as ambiguous losses.

Although BEP-TG consists of components with proven effectiveness in the treatment of PTSD, PCBD, and MDD, the efficacy of the full BEP-TG protocol in reducing symptoms of these disorders following traumatic loss remains to be established. Future research should elucidate treatment efficacy in various groups, such as disaster-stricken populations, refugees, groups at risk of profession-related traumatic loss (e.g., police officers, military veterans), and suicide bereaved persons. Future studies may also explore which symptoms are most likely to resolve during specific phases of the treatment, thus furthering our understanding of how modification of the cognitive, attachment, and stress processes involved in traumatic grief contributes to recovery.

## Supplementary Material

Brief Eclectic Psychotherapy for Traumatic Grief (BEP-TG): toward integrated treatment of symptoms related to traumatic loss

Brief Eclectic Psychotherapy for Traumatic Grief (BEP-TG): toward integrated treatment of symptoms related to traumatic loss

Brief Eclectic Psychotherapy for Traumatic Grief (BEP-TG): toward integrated treatment of symptoms related to traumatic loss

Brief Eclectic Psychotherapy for Traumatic Grief (BEP-TG): toward integrated treatment of symptoms related to traumatic loss

Brief Eclectic Psychotherapy for Traumatic Grief (BEP-TG): toward integrated treatment of symptoms related to traumatic loss
