# Supplementary material for: Brief Eclectic Psychotherapy for Traumatic Grief (BEP-TG): toward integrated treatment of symptoms related to traumatic loss
Source: Eur J Psychotraumatol. 2015 Jul 6;6:10.3402/ejpt.v6.27324. doi: 10.3402/ejpt.v6.27324 (PMC4495623; doi:10.3402/ejpt.v6.27324)
Supplement: Brief Eclectic Psychotherapy for Traumatic Grief (BEP-TG): toward integrated treatment of symptoms related to traumatic loss [file EJPT-6-27324-s001.pdf]

Psicoterapia ecléctica breve para el duelo traumático (PEB-DT)

Geert Edzko Smid, Rolf J Kleber, Simone M de la Rie, Jannetta B.A. Bos, Berthold P.R. Gersons, Paul A. Boelen

Antecedentes: Los acontecimientos traumáticos, tales como desastres, accidentes, guerras o violencia criminal, a menudo van acompañados de la pérdida de seres queridos y por tanto pueden dar lugar a un duelo traumático. El duelo traumático hace referencia al diagnóstico clínico de trastorno por duelo complejo persistente (TDCP) con (síntomas de) trastorno de estrés postraumático (TEPT) comórbido y/o trastorno depresivo mayor (TDM) después de haberse enfrentado a una pérdida traumática. Los sobrevivientes de trauma, que con frecuencia provienen de diferentes entornos culturales, suelen haber experimentado numerosas pérdidas, además de pérdidas ambiguas (familiares o amigos desaparecidos). Los tratamientos actuales con base empírica para el TEPT no se centran en el duelo traumático.

Objetivo: Desarrollar un tratamiento para el duelo traumático que combine intervenciones de tratamiento para TEPT y TCDP y que pueda acomodar los aspectos culturales del duelo.

Método: Para darle un razonamiento al tratamiento, proponemos un modelo de estrés cognitivo del duelo traumático. Basándonos en este modelo y en los tratamientos actuales con base empírica para el TEPT y el duelo traumático, desarrollamos la psicoterapia ecléctica breve para el duelo traumático (PEB-DT) , para tratar pacientes con duelo traumático. Presentamos el tratamiento junto con un caso clínico.

Resultados: Los procesos que contribuyen al duelo traumático incluyen la integración inadecuada del recuerdo de la pérdida traumática, la valoración negativa de la pérdida traumática, la sensibilidad a disparadores similares y factores de estrés nuevos y el intento de evitar el malestar. La PEB-DT trabaja sobre estos procesos. El protocolo de la PEB-DT consiste en cinco partes que han probado su eficacia en el tratamiento de el TCDP, el TEPT y el TDM: información y motivación, exposición centrada en el duelo, memorabilia y tareas por escrito, darle sentido y activación y un ritual de despedida.

Conclusión: Cortada a medida para cubrir las necesidades de los sobrevivientes del trauma, la PEB-DT puede utilizarse para tratar síntomas de duelo traumático relacionados con múltiples pérdidas y pérdidas ambiguas, así como los aspectos culturales del duelo a través de sus diferentes componentes.

Palabras clave: duelo, trauma, TEPT, depresión, cognitivo, apego, psicoterapia ecléctica breve, refugiado, dolor

Name of translator: Miriam Ramos Morrison

Citation: European Journal of Psychotraumatology 2015, **6**: 27324 - http://dx.doi.org/10.3402/ejpt.v6.27324
