# Supplementary material for: Brief Eclectic Psychotherapy for Traumatic Grief (BEP-TG): toward integrated treatment of symptoms related to traumatic loss
Source: Eur J Psychotraumatol. 2015 Jul 6;6:10.3402/ejpt.v6.27324. doi: 10.3402/ejpt.v6.27324 (PMC4495623; doi:10.3402/ejpt.v6.27324)
Supplement: Brief Eclectic Psychotherapy for Traumatic Grief (BEP-TG): toward integrated treatment of symptoms related to traumatic loss [file EJPT-6-27324-s002.pdf]

Brief Eclectic Psychotherapy for Traumatic Grief (BEP-TG)

Geert Edzko Smid, Rolf J Kleber, Simone M de la Rie, Jannetta B.A. Bos, Berthold P.R. Gersons, Paul A. Boelen

Hintergrund: Traumatische Ereignisse wie Katastrophen, Unfälle, Krieg oder kriminelle Gewalt gehen oft mit dem Verlust nahestehender Menschen einher und können dann traumatische Trauer hervorrufen. Der Begriff traumatische Trauer bezieht sich auf die klinische Diagnose einer persistent complex bereavement disorder (PCBD) mit komorbiden Symptomen einer posttraumatischen Belastungsstörung (PTBS) und/oder einer Major Depression Disorder (MDD) als Folge eines traumatischen Verlustes. Oft haben Überlebende eines Traumas, die häufig unterschiedliche kulturelle Hintergründe haben, mehrfache Verluste oder einen ungewissen Verlust (vermisste Familienmitglieder oder Freunde) erlitten. Der Fokus von aktuellen evidenzbasierten PTBS-Behandlungen liegt nicht auf traumatischer Trauer.

Zielsetzung: Eine Behandlung für traumatische Trauer entwickeln, die jene Behandlungs-maßnahmen für PTBS und PCBD kombiniert, die an die kulturellen Aspekte von Trauer angepasst sind.

Methode: Zur Begründung einer Behandlung schlagen wir ein kognitives Stressmodell der traumatischen Trauer vor. Basierend auf diesem Modell und auf vorliegenden evidenzbasierten Behandlungsleitlinien für PTBS und komplizierte Trauer entwickelten wir die Brief Eclectic Psychotherapy for Traumatic Grief (BEP-TG). Diese dient der Behandlung von Patienten mit traumatischer Trauer, welche gemeinsam mit einer Fallvignette präsentiert wird.

Ergebnisse: Zu den Prozessen, die zu traumatischer Trauer beitragen, zählen: inadäquate Integration der Erinnerung an den traumatischen Verlust, negative Bewertung des traumatischen Verlusts, Sensitivität gegenüber entsprechenden Triggern und neuen Stressoren und der Versuch, Stress zu vermeiden. Die BEP-TG zielt auf diese Prozesse ab. Das Protokoll der BEP-TG besteht aus fünf Teilen mit erwiesener Effektivität in der Behandlung von PCBD, PTBS und MDD: Information und Motivation, trauerfokussierte Exposition, Erinnerungsobjekte und Schreibübungen, Sinnfindung und Aktivierung sowie einem Abschiedsritual.

Schlussfolgerung: Die BEP-TG, abgestimmt auf die Bedürfnisse von Traumaüberlebenden, kann zur Behandlung von Symptomen traumatischer Trauer verwendet werden, die in Verbindung mit mehrfachen Verlusten oder einem ungewissen Verlust stehen. Zudem zielt die BEP-TG anhand ihrer verschiedenen Komponenten auf kulturelle Aspekte schmerzlicher Verluste ab.

Keywords: Trauer, Trauma, PTBS, Depression, kognitiv, Bindung, Brief Eclectic Psychotherapy, Flüchtlinge, Verlust

Name of translator: LD

Citation: European Journal of Psychotraumatology 2015, **6**: 27324 - http://dx.doi.org/10.3402/ejpt.v6.27324
