# Supplementary material for: Brief Eclectic Psychotherapy for Traumatic Grief (BEP-TG): toward integrated treatment of symptoms related to traumatic loss
Source: Eur J Psychotraumatol. 2015 Jul 6;6:10.3402/ejpt.v6.27324. doi: 10.3402/ejpt.v6.27324 (PMC4495623; doi:10.3402/ejpt.v6.27324)
Supplement: Brief Eclectic Psychotherapy for Traumatic Grief (BEP-TG): toward integrated treatment of symptoms related to traumatic loss [file EJPT-6-27324-s003.pdf]

创伤性悲痛的简明折衷心理治疗(BEP-TG)

Authors: Geert Edzko Smid, Rolf J Kleber, Simone M de la Rie, Jannetta B.A. Bos, Berthold P.R. Gersons, Paul A. Boelen

背景：创伤性事件如灾难、事故、战争、暴力犯罪经常伴随亲人的去世，并因此可能带来创伤性悲痛。创伤性悲痛涉及持续复杂丧亲障碍（PCBD）的临床诊断，伴随由于创伤性丧亲而产生的创伤后应激障碍(PTSD)和/或重性抑郁障碍(MDD)症状。来自不同文化背景的创伤幸存者时常经历多重丧失，诸如丧失家庭成员或朋友。目前关于 PTSD 的循证治疗并不针对创伤性悲痛。

目的：发展一种对于 PTSD、PCBD 及创伤性悲痛的联合治疗干预，这种干预可能考虑到文化在悲痛的影响。

方法：我们提出了一个创伤性悲痛的认知应激模型作为治疗的理论依据。基于该应激模型以及现有治疗 PTSD 和复杂悲痛的证据，我们针对创伤性悲痛的患者发展了简明折衷心理治疗（BEP-TG），并包括了一个病例来介绍这种治疗方法。

结果：引起创伤性悲痛的过程因素包括：涉及创伤性丧失的不恰当的记忆整合、对创伤性丧失持负性评价、对新应激和类似刺激敏感、对痛苦进行回避。BEP-TG 治疗就针对这些过程。BEP-TG 治疗方案包含 5 个部分，这些部分已经被证明在 PCBD、PTSD、MDD 的治疗有效，即信息和动机、聚焦于悲痛的暴露、备忘录和书写作业、找到意义和激活、仪式化告别。

结论：通过调整不同部分来适应创伤幸存者的需求，BEP-TG 可以用于处理创伤性悲痛症状，即使这些症状与多重丧失、不明确的丧失有关，或受到文化方面影响。

Key words: 悲痛、创伤、PTSD、抑郁、认知、依恋、简明折衷心理治疗、难民、丧亲

Name of translator: Xuan JU, Zhonglin Tan

Citation: European Journal of Psychotraumatology 2015, **6**: 27324 - http://dx.doi.org/10.3402/ejpt.v6.27324
