# Supplementary material for: Brief Eclectic Psychotherapy for Traumatic Grief (BEP-TG): toward integrated treatment of symptoms related to traumatic loss
Source: Eur J Psychotraumatol. 2015 Jul 6;6:10.3402/ejpt.v6.27324. doi: 10.3402/ejpt.v6.27324 (PMC4495623; doi:10.3402/ejpt.v6.27324)
Supplement: Brief Eclectic Psychotherapy for Traumatic Grief (BEP-TG): toward integrated treatment of symptoms related to traumatic loss [file EJPT-6-27324-s004.pdf]

Ogólna eklektyczna terapia traumatycznego żalu

Geert Edzko Smid, Rolf J Kleber, Simone M de la Rie, Jannetta B.A. Bos, Berthold P.R. Gersons, Paul A. Boelen

Wprowadzenie: Zdarzenia traumatyczne, jak katastrofy, wypadki, wojny czy napady często wiążą się z utratą ukochanej osoby i mogą pociągac za sobą traumatyczny żal. Trauamatyczny żal przejawia cechy klinicznej diagnozy złożonego zaburzenia uporczywej żałoby, z towarzyszącymi symptomami PTSD oraz wielkiej depresji. Osoby po przeżyciach traumatycznych pochodzące z odmiennych kultur mogą doswiadczyć wielokrotnej straty. Obecne, oparte na wynikach badań empirycznych studia nad PTSD nie skupiają się jednak na traumatycznym żalu.
Cel: Celem tej pracy było opracowanie metody terapii dla osób doświadczających traumatycznego żalu oraz współtowarzyszących objawów PTSD i żałoby przy uwzględnieniu specyfiki różnorodności kulturowej.
Metoda: W niniejszej pracy bazowaliśmy na poznawczym modelu stresu towarzyszącemu traumatycznemu żalowi. Na podstawie tego modelu, jak również odwołując się do wybranych technik terapii PTSD sformułowalismy podstawy pod ogólną eklektyczną terapię traumatycznego żalu (ang. brief eclectic psychotherapy for traumatic grief).
Wyniki: Mechanizm odpowiedzialny za wykształcenie się symptomów traumatycznego żalu obejmuje dysfunkcjonalne mechanimzy pamięciowe, negatywne pobudzenie oraz nadmierną wrażliwość na wszelkie bodźce przypominające utracony obiekt.
Konkulzje: Zaproponowana forma terapii traumatycznego żalu może nieśc ulge osobom doświadczającym tego klinicznego zjawiska. Istotne jest również to, że zaproponowana przez nas terapia uwzględnia różnice międzykulturowe.

Słowa kluczowe: żal, trauma, PTSD, depresja, poznawczy, przywiązania, ogólna terapia eklektyczna, uchodziec, utrata

Name of translator: Marcin Rzeszutek, University of Finance and Management in Warsaw, Poland

Citation: European Journal of Psychotraumatology 2015, 6: 27324 - http://dx.doi.org/10.3402/ejpt.v6.27324
