# Supplementary material for: Brief Eclectic Psychotherapy for Traumatic Grief (BEP-TG): toward integrated treatment of symptoms related to traumatic loss
Source: Eur J Psychotraumatol. 2015 Jul 6;6:10.3402/ejpt.v6.27324. doi: 10.3402/ejpt.v6.27324 (PMC4495623; doi:10.3402/ejpt.v6.27324)
Supplement: Brief Eclectic Psychotherapy for Traumatic Grief (BEP-TG): toward integrated treatment of symptoms related to traumatic loss [file EJPT-6-27324-s005.pdf]

Brief Eclectic Psychotherapy for Traumatic Grief (BEP-TG): toward integrated treatment of symptoms related to traumatic loss

Geert E. Smid, Rolf J. Kleber, Simone M. de la Rie, Jannetta B. A. Bos, Berthold P. R. Gersons and Paul A. Boelen

Samenvatting

Achtergrond: Traumatische gebeurtenissen zoals rampen, ongevallen, oorlog of crimineel geweld gaan vaak gepaard met het verlies van dierbaren, en kunnen dan aanleiding geven tot traumatische rouw. Traumatische rouw verwijst naar een klinische diagnose van persisterende complexe rouwstoornis (PCRS) met comorbide (symptomen van) posttraumatische stress-stoornis (PTSS) en / of depressieve stoornis na confrontatie met een traumatisch verlies. Overlevenden van traumatische gebeurtenissen, doorgaans met uiteenlopende culturele achtergronden, hebben vaak meerdere verliezen ervaren evenals ambigu verlies (vermiste familieleden of vrienden). Huidige evidence-based behandelingen voor PTSS zijn niet gericht op traumatische rouw.

Doel: Het ontwikkelen van een behandeling voor traumatische rouw door combineren van behandelinterventies voor PTSS en PCRS, die ruimte biedt voor culturele aspecten van rouw.

Methode: Als rationale voor behandeling wordt een cognitief stress-model van traumatische rouw voorgesteld. Op basis van dit model en bestaande evidence based behandelingen voor PTSS en gecompliceerde rouw, ontwikkelden wij Beknopte Eclectische Psychotherapie voor Traumatische Rouw (BEP-TG) voor de behandeling van patiënten met traumatische rouw. De behandeling wordt gepresenteerd samen met een casusvignet.

Resultaten: Processen die bijdragen aan traumatische rouw omvatten onvoldoende integratie van herinneringen aan het traumatische verlies, negatieve interpretatie van het traumatische verlies, gevoeligheid voor triggers en nieuwe stressoren en pogingen om emotionele pijn te vermijden. BEP-TG richt zich op deze processen. Het BEP-TG-protocol bestaat uit vijf onderdelen met bewezen effectiviteit in de behandeling van PCRS, PTSS en depressie: informatie en motivatie, rouwgerichte exposure, memorabilia en schrijfopdrachten, betekenisgeving en activering, en een afscheidsritueel.

Conclusie: Afgestemd op de behoeften van overlevenden na traumatisch verlies, kunnen de verschillende onderdelen van BEP-TG worden gebruikt voor de behandeling van traumatische rouw symptomen die verband houden met meerdere verliezen en ambigu verlies, rekening houdend met culturele aspecten van rouw.

Sleutelwoorden: rouw, trauma, PTSS, depressie, cognitief, gehechtheid, beknopte eclectische psychotherapie, vluchteling, verlies

Citation: European Journal of Psychotraumatology 2015, 6: 27324 - http://dx.doi.org/10.3402/ejpt.v6.27324
